# Full Spectrum Flow Cytometry as a Powerful Technology for Cancer Immunotherapy Research

**DOI:** 10.3389/fmolb.2020.612801

**Published:** 2021-01-29

**Authors:** Diana L. Bonilla, Gil Reinin, Edmond Chua

**Affiliations:** Cytek Biosciences, Fremont, CA, United States

**Keywords:** full spectral flow cytometry, immunomonitoring, cancer immunotherapy, immunology, cytometry

## Abstract

The Nobel Prize-deserving concept of blocking inhibitory pathways in T cells, to unleash their anti-tumoral capacity, became one of the pillars of cancer treatment in the last decade and has resulted in durable clinical responses for multiple cancer types. Currently, two of the most important goals in cancer immunotherapy are to understand the mechanisms resulting in failure to checkpoint blockade and to identify predictive immunological biomarkers that correlate to treatment response, disease progression or adverse effects. The identification and validation of biomarkers for routine clinical use is not only critical to monitor disease or treatment progression, but also to personalize and develop new therapies. To achieve these goals, powerful research tools are needed. Flow cytometry stands as one of the most successful single-cell analytical tools used to characterize immune cell phenotypes to monitor solid tumors, hematological malignancies, minimal residual disease or metastatic progression. This technology has been fundamental in diagnosis, treatment and translational research in cancer clinical trials. Most recently, the need to evaluate simultaneously more features in each cell has pushed the field to implement more powerful adaptations beyond conventional flow cytometry, including Full Spectrum Flow Cytometry (FSFC). FSFC captures the full emission spectrum of fluorescent molecules using arrays of highly sensitive light detectors, and to date has enabled characterization of 40 parameters in a single sample. We will summarize the contributions of this technology to the advancement of research in immunotherapy studies and discuss best practices to obtain reliable, robust and reproducible FSFC results.

## Introduction

According to the World Health Organization, Cancer is the second leading cause of disease burden and mortality in the world, and strategies for cancer prevention, diagnosis, and treatment are still a global effort (Mattiuzzi and Lippi, 2019). Immuno-oncology (IO) is the study of the immune system functionality against cancer and the development of treatments that improve the ability of the immune system to fight the disease (Guo et al., 2019). The enhanced response against cancer is achieved by improving the immune cell recruitment into the tumor microenvironment and/or the recognition and destruction of cancer cells by immune cells (Sharma and Allison, 2015). Cancer immunotherapy has become the standard of care for multiple tumor types at diverse disease stages, providing better survival and reduced disease recurrences (Sharma et al., 2011). Multiple clinical trials with successful implementation of IO treatments are being conducted to evaluate the efficacy of FDA-approved treatments in multiple cancer types, to implement combinatorial therapies, to gain mechanistic insight for new drug development or to identify immune predictors of clinical response (Wei et al., 2018).

Biomarker discovery in clinical trials requires robust and reproducible technologies to characterize immune cells, with evaluation of lineage-defining proteins, activation/exhaustion markers, cytokines, transcription factors, immune checkpoints and cell functionality. Flow cytometry (FC) enables high-throughput profiling of single cells in suspension labeled with fluorochromes upon laser illumination, and it has become a standard tool to monitor dynamic changes in the lymphoid and myeloid compartments of the immune response in IO (Allison, 2015). This technology has been implemented for multiple cancer applications: DNA content evaluation to study cell cycle and ploidy, cell immunophenotyping in hematological malignancies as a diagnostic tool, identification of residual tumor cells to study minimal disease or characterization of circulating tumor cells to study metastatic events (Cossarizza et al., 2019). Ability to sort cells after immunophenotyping provides downstream functional and DNA/RNA analysis capabilities, giving a more complete picture of cancer immunobiology and pathogenesis.

The development of more advanced technologies in the last decade, including mass cytometry (Spitzer and Nolan, 2016), imaging flow cytometry (Basiji, 2016), genomic cytometry (Robinson, 2004; Stoeckius et al., 2017) and spectral cytometry (Robinson, 2004; Nolan et al., 2013), has expanded our ability to study immune responses, by characterizing more cellular parameters simultaneously in single cells with higher resolution than ever before. This review provides a critical overview of the FSFC contributions to the IO field, as well as the guidelines to generate high quality data for immune monitoring using this technology.

## Cancer Immunotherapy

The immune system can target and destroy cancer cells, but tumors can grow if that response is evaded. IO is the study, development and evaluation of treatments that exploit the immune system to fight cancer. IO-based therapies cover different approaches to boost the immune response in cancer patients, that range from activation of effector cells, vaccination with tumor antigens, administration of oncolytic viruses, blockage of inhibitory pathways or immunosuppressive mechanisms, use of adoptive chimeric antigen receptor T-cell therapy; to amplification of protective pathways (Allison, 2015). The clinical efficacy of immunotherapy led to the US FDA approval of ipilimumab in 2010, the first checkpoint inhibitor, a fully human monoclonal antibody targeting the inhibitory CTLA-4 protein in T cells. Ipilimumab was the first cancer immunotherapy to demonstrate durable clinical responses, increased long term-survival and manageable toxicity in metastatic melanoma patients (Hodi et al., 2010).

Since then, a plethora of agents that unleash the anti-tumoral capacity of the immune system, including checkpoint inhibitors, adoptive cell therapies, and cancer vaccines, have been approved with benefit across many tumor types and stages. However, immunotherapy is not successful in all cancer patients given the dynamics of the immune system and the diversity of cell populations infiltrating the tumor microenvironment. Better characterization of the immune responses in patients receiving immunotherapy is needed to fully understand the mechanisms of drug resistance (Sharma and Allison, 2015). There is a clear interest in identifying and validating predictive biomarkers to guide treatment decisions and to select personalize IO regimens based on clinical outcome (Fox et al., 2011). This immune monitoring is also critical to identify signatures associated to off-target or undesirable responses in patients treated with immunotherapy and to design novel immune therapies (Yuan et al., 2016).

## Full Spectrum Flow Cytometry

IO focuses on how cells in the immune system respond to cancer, so the use of single cell analytical technologies is critical to define patient immunological profiles and guide treatment decisions. FC characterizes physical and fluorescent properties of cells in suspension by using fluorochrome-conjugated antibodies to measure proteins expressed by distinct immune cell subpopulations (Cossarizza et al., 2019). Immunophenotyping enables lineage definition and differentiation state of cell populations, critical for classification of hematological malignancies and myelodysplastic syndromes and for monitoring clinical response to treatment or recurrences (Heel et al., 2013). FC facilitates the identification of tumor circulating cells that survive after treatment given their correlation with higher relapse and poor survival rates (Irish and Doxie, 2014). Furthermore, this technology enables the evaluation of cell proliferation and DNA ploidy analysis and the identification of rare events, by the characterization of millions of cells in a short time (Maecker and Harari, 2015; Cunningham et al., 2019). Compared to other single cell analytical technologies, FC is often chosen over others for its ability to analyze cells faster at tens of thousands of cells per second, relatively low sample volume requirements, improved maintenance costs, shorter and easier sample preparation and instrument set-up protocols (Barone et al., 2020; Ferrer-Font et al., 2020a; Niewold et al., 2020). In addition, FSFC equipped with sorting capability will allow to sort cells for down-stream analyses, supporting genomics technology and gene profiling of cells.

Flow cytometry has rapidly evolved over the past few decades after being introduced in 1969 by Göhde et al. (Robinson, 2019). Conventional FC uses band pass filters and light detectors to capture the peak of fluorescence emission, having a single dedicated detector and filter per fluorochrome. This approach has been traditionally used, but its limited resolution and multiplexing capabilities moved the field towards the development of more powerful instrumentation with higher sensitivity optics and cost-effectiveness in mind. All cytometers measure incident photons, derived from fluorescent proteins, fluorochromes or fluorescent dyes, but the challenge is to make sure those photons are efficiently captured and resolved from noise. Spectral cytometry measures the complete fluorescence spectrum of individual cells, capturing in multiple detectors the full emission across the entire wavelength range of visible light (Nolan and Condello, 2013). Spectral flow cytometry was first demonstrated by Dr. Paul Robinson at Purdue University in 2004 (Robinson, 2004). The first commercial instrument was launched by Sony Biotechnology in 2012, using prisms along with PMT detectors to collect and amplify light beyond the capability of conventional flow cytometers. Initial advantages of these early spectral flow cytometers over conventional flow cytometers were the ability to use new combinations of fluorochromes together and the ability to measure and extract autofluorescence contributions from unstained cells from the total fluorescence signal in stained cells.

**FIGURE 1:**
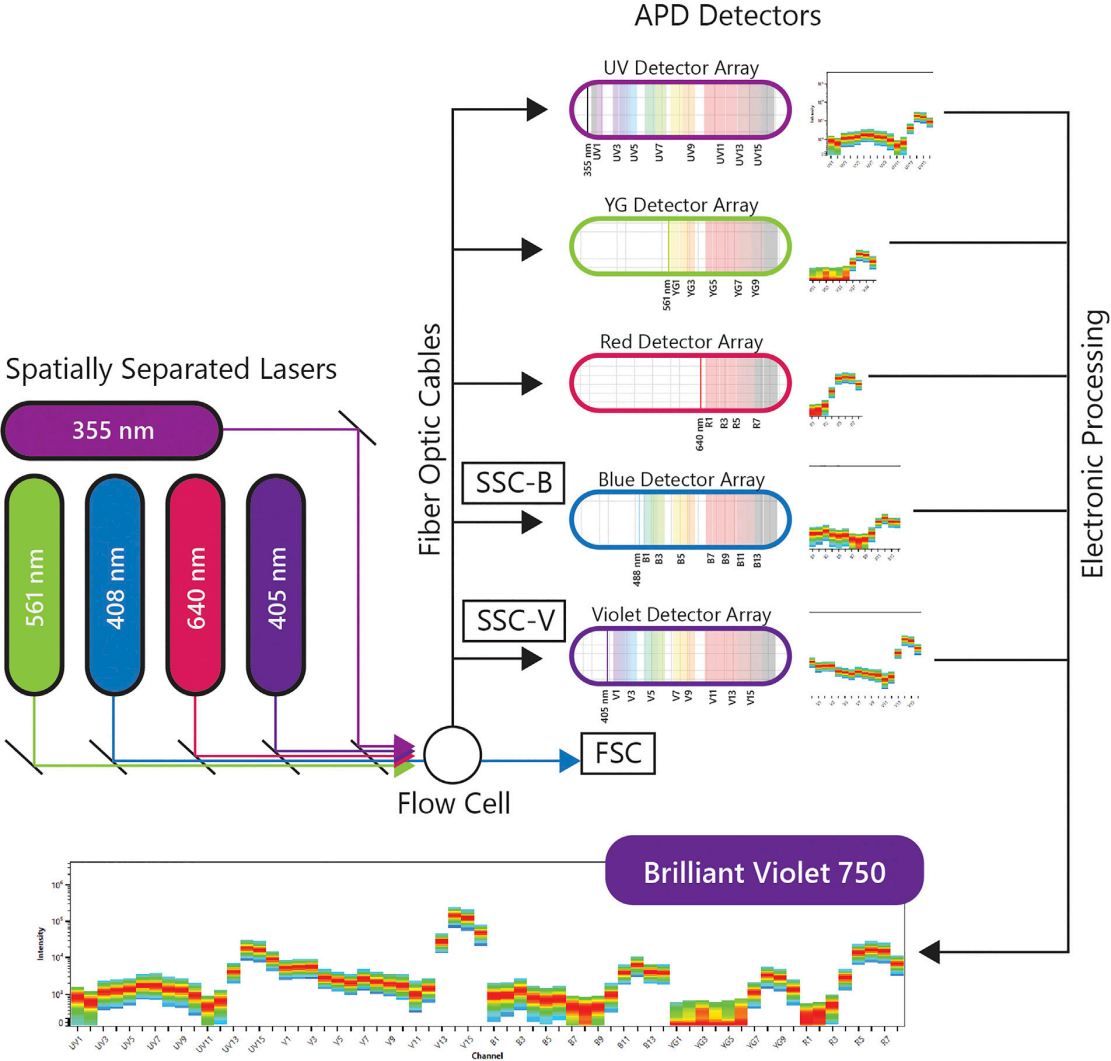
Full Spectral Flow Cytometry Diagram. FSFC captures the full emission spectrum of every fluorescent molecule across a defined range of wavelengths, by using highly sensitive light detectors, and enables high-throughput characterization of beyond 40 parameters at a single cell level from one sample.

A more recent implementation of spectral flow cytometry, Full Spectrum Flow Cytometry (FSFC), was introduced by Cytek Biosciences in 2017 and was rated one of the top immunotherapy companies of 2020 by Pharma Tech Outlook. The immunological evaluations using the Cytek’s FSFC approach will be the center of discussion in this review. Cytek’s high end FSFC system, the Cytek Aurora, has up to 5 lasers, 3 scatter detectors and 64 fluorescence detectors. The uniqueness of FSFC is in the optical design using improved semiconductor detectors and computational analytics for full-spectral measurement of multiple dyes emitting in the 360–830 nm wavelength range. Each laser has one detector array and each array has an optical filter based coarse wavelength division multiplexing demultiplexer assembly to disperse the emitted light across an array of detectors, without using gratings or prisms as dispersive elements. This system uses avalanche photo diodes (APDs) as light detectors. APDs capture light emission with narrower bandwidths, lower electronic noise and better quantum efficiency, especially in red and near-infrared wavelengths, which correlates with better resolved dim and rare cell populations in increasingly complex multicolor samples. The electronic signal is collected, amplified and digitized by complex electronics, and then the multicolor data is decomposed into different channels to analyze the contribution of each fluorochrome into fully stained samples. A schematic of the FSFC components is shown in Figure 1.

By measuring the full spectrum of light emissions from each cell, FSFC can differentiate combinations of fluorophores that conventional systems cannot. This in turn has increased flexibility in application design and enabled the development of highly complex multicolor panels for comprehensive immunophenotypic analysis by flow cytometry. Recently, Park et al. described the characterization of hematopoietic cells by FSFC with quantification of 40 fluorescent parameters per cell (Park et al., 2020). High resolution for every cell subset identified was achieved for both myeloid and lymphoid populations. Another recent study by Niewold et al. evaluated the quality of immunophenotyping between conventional and spectral cytometers. The full spectrum characterization of every fluorescent dye, the use of sensitive APD detectors and the autofluorescence extraction translated into better separation of overlapping fluorochromes, reduced spreading error and outstanding population resolution in multiple datasets evaluated (Niewold et al., 2020). FSFC immunophenotyping data is highly comparable with mass cytometry, another well-established technique for immune monitoring (Barone et al., 2020; Ferrer-Font et al., 2020a). Both studies reported better cell recovery and faster acquisition times using spectral approaches. FSFC is ideal for characterization of low-frequency populations and samples with low cell numbers, interrogating millions of cells in shorter time. FSFC has been approved by the CE marking for in vitro diagnostic devices for its clinical diagnostic use in Europe.

The availability of unique fluorochromes and the spillover between fluorochromes are still technical limitations for this technology. In the past year, an increasing number of new dyes with better and different emission profiles have been released, allowing investigators to build larger panels and measure more proteins simultaneously in their samples. The FSFC developments in instrumentation and reagents, have been accompanied by impressive advancements in data analysis computational tools to explore the high-dimensional datasets generated (Ferrer-Font et al., 2020a; Fox et al., 2020; Park et al., 2020; Silvin et al., 2020). Advanced computational algorithms facilitate deep and objective exploration of highly complex datasets, with visualization of specific cell clusters and their association with clinical variables. All these improvements in cytometry methodologies have extended the utility and usefulness of flow cytometry in IO research, enabling in-depth characterization of immunological profiles and revealing new insights into cancer biology (Bendall and Nolan, 2012; Irish and Doxie, 2014).

Conventional manual gating analysis of high-dimensional data is subjective, time-consuming and does not capture the complexity of multidimensional datasets. Multiple options, including plug-ins embedded in commercial software, cloud-based platforms or scripts implemented using programing languages, are now available to explore the dataset in a more comprehensive and objective manner to understand the complexity of the multiple cell subsets evaluated. FSFC data has driven the design of new computational tools and algorithms for data mining, facilitating data visualization and extraction of statistical correlations. The study by Fox et al. developed Cyto-Feature Engineering, a new R-based pipeline to identify immune populations. This tool uses Fluorescence Minus One (FMO) controls to set cutoff thresholds for positive and negative marker expression. The data is then organized by lineages, statistically correlated with other variables and visualized using heatmaps of cell phenotypes (Fox et al., 2020). Barone et al. tested the Tracking Responders Expanding (T-REX), a new machine learning algorithm to evaluate immune changes in response to disease. Using this immune monitoring automated toolkit, this study identified that CD4+ T cells expand rapidly in response to immunotherapy (Barone et al., 2020). Other studies have implemented algorithms for dimensionality reduction, such as (t-distributed stochastic neighbor embedding (t-SNE), uniform manifold approximation and projection (UMAP) and clustering tools, including FlowSOM and PhenoGraph to characterize immune cell subsets identified by FSFC (Ferrer-Font et al., 2020a; Park et al., 2020; Silvin et al., 2020).

## FSFC Contributions to Cancer Immunotherapy Research

FSFC has facilitated the study of the complexity of tumor immune infiltrates and biomarker exploration for clinical response correlations. The findings by Ng et al. described the value of CD38 as a positive predictive biomarker in macrophages infiltrating hepatocellular carcinoma in patients receiving immune-checkpoint therapy (anti PD-1/PD-L1). FSFC was used in this study to validate CD38 expression in macrophages, using CD14, CD16, CD11c, CD68, CD11b and HLA-DR for myeloid lineage and cell subset definition (Ng et al., 2020). Bauman et al. investigated changes in immune signatures in a phase I study of an anti-HGF antibody and an anti-EGFR antibody in patients with cetuximab-resistant, recurrent/metastatic squamous cell carcinoma. An increase in peripheral T cells, particularly a CD8^+^ subset, was associated with treatment response (Bauman et al., 2020). Mukherjee et al identified CCL5 as a poor prognostic blood biomarker associated with lower overall survival in patients with advanced pancreatic cancer treated with anti-PD-1. The authors hypothesize that a CCL5 inhibitor in combination with anti-PD-1 could overcome resistance to treatment. One last example, the translational analysis results from the SCALOP multi-center phase II trial were presented at the ASCO Conference this year (Mukherjee et al., 2020). The study by Zhang et al. provided a ranking of the sensitivity of human cancers to anti-CTLA-4 antibodies. They tested a new generation of antibodies with reduced induction of adverse effects and enhanced antibody-dependent cell-mediated cytotoxicity. The non-small cell lung carcinoma (NSCLC) was predicted to be highly responsive to anti-CTLA-4 antibodies. FSFC was used to evaluate human NSCLC-infiltrating T cells, finding depletion of and regulatory T cells in the tumor, a hallmark of efficient CTLA-4 blockade. These findings suggest that NSCLC will likely respond to these new newly designed anti-CTLA-4 antibodies (Zhang et al., 2020).

Other studies have implemented FSFC to characterize tumor-infiltrating immune cells in response to treatment using animal models. Johnson et al. showed that MHC Class II regulates the infiltrating T cells and the subsequent response to anti-PD-1 treatment in lung adenocarcinoma. The authors demonstrated that over-expression of CIITA, a master regulator of the MHCII pathway, increased T cell infiltration and tumor response to anti-PD-1 therapy (Johnson et al., 2020). Resistance to immune checkpoint blockade is likely due to compensatory upregulation of additional inhibitory receptors. The study by Yang et al. described that Tim-3, an inhibitory checkpoint, was up-regulated in regulatory T cells, CD4+ and CD8+ T cells, dendritic cells and macrophages in the tumor microenvironment. Treatment with Tim-3, PD-1, and Lag3 antibodies resulted in higher cytotoxic activity of infiltrating CD8+ T cells, tumor regression and higher survival in MC38 tumor-bearing mice (Yang et al., 2020).

FSFC has been a powerful tool for immunological evaluations when exploring new cancer therapeutic targets. Patients with T cell lymphoma have limited treatment options because their cancer cells evade apoptosis by Bcl-xl upregulation. PROTAC, a proteolysis-inducing compound, targets Bcl-xl for degradation as a potent anti-tumoral agent. This intervention reduced disease progression and increased survival in a TCL PDX mouse model dependent on both Bcl-2 and Bcl-xl. (He et al., 2020; Zhang et al., 2020). The same PROTAC approach has been used to reduce platelet toxicity of navitoclax, a known Bcl-2 and Bcl-xl dual inhibitor (He et al., 2020). In these studies, FSFC was implemented to evaluate T cells, B cells, myeloid cells, hematopoietic progenitors and hematopoietic stem cells in bone marrow. Another study explored the use extracellular cGAMP, an immune transmitter produced by cancer cells in response to dsDNA, in radiation-induced protective responses. In mouse tumors, depletion of cGAMP decreased tumor infiltration by immune cells and inhibited the protective radiation effect, while increase of cGAMP synergized with radiation to delay tumor growth (Carozza et al., 2020).

Immune characterization is also a critical step in cancer vaccine development. The FSFC technology enables both structural and functional characterization of immune cells for vaccine studies, including dendritic cell antigen presentation and migration to lymph node and polarization and proliferation of effector T cells, by quantifying the expression of multiple transcriptional factors T-bet, Gata3, RORγt, Bcl6, Foxp3. Si et al. designed an adjuvant-free nano vaccine that induces in situ lung dendritic cell activation and accumulation of antigen-specific Th17 T cells in lymph node and lung, with subsequent induction of a protective response (Si et al., 2020). The study by Bommireddy et al showed that a vaccine using tumor membrane vesicles (TMV), enhances the efficacy of anti-PD1 immune checkpoint treatment in inhibitor-resistant squamous cell carcinoma. Membrane vesicles are prepared from tumors and then incorporated with immunostimulatory molecules by protein transfer to generate the TMV vaccine. TMV inhibited tumor growth and improved the survival of mice challenged with SCCVII tumor cells (Bommireddy et al., 2020). Cellular senescence is characterized by cell-cycle arrest that prevents tumor cell expansion. The study by Amor C. et al identified a urokinase-type plasminogen activator receptor (uPAR), a protein induced during senescence. uPAR-specific chimeric antigen receptor (CAR) T cells target and destroy senescent cells, extending the survival of mice with lung adenocarcinoma. These results discovered the therapeutic potential of senolytic CAR T cells (Amor et al., 2020).

Natural killer (NK) cells play a role in tumorigenesis and multiple treatment alternatives are focused in this cell population. FSFC helps to characterize structural and functional features in NK cells. The study by Ng et al. showed that the natural killer cell granule protein 7 (NKG7), a protein expressed by NK cells that controls degranulation, is important to prevent cancer growth and dissemination (Ng 2020). Wilk et al. described the use of non-viral transporters to efficiently transfect primary human NK cells with mRNA without the need of NK cell activation and preserving cell cytotoxicity and viability for therapeutic applications (Wilk et al., 2020). Finally, Shissler S et al. demonstrated that a subset of thymic resident NKT cells displays differential requirements for CD28 co-stimulation during antigenic activation. Cell proliferation was impaired when CD28 engagement was blocked, but unaffected by CTLA-4 activation or blockade (Shissler et al., 2020).

**FIGURE 2:**
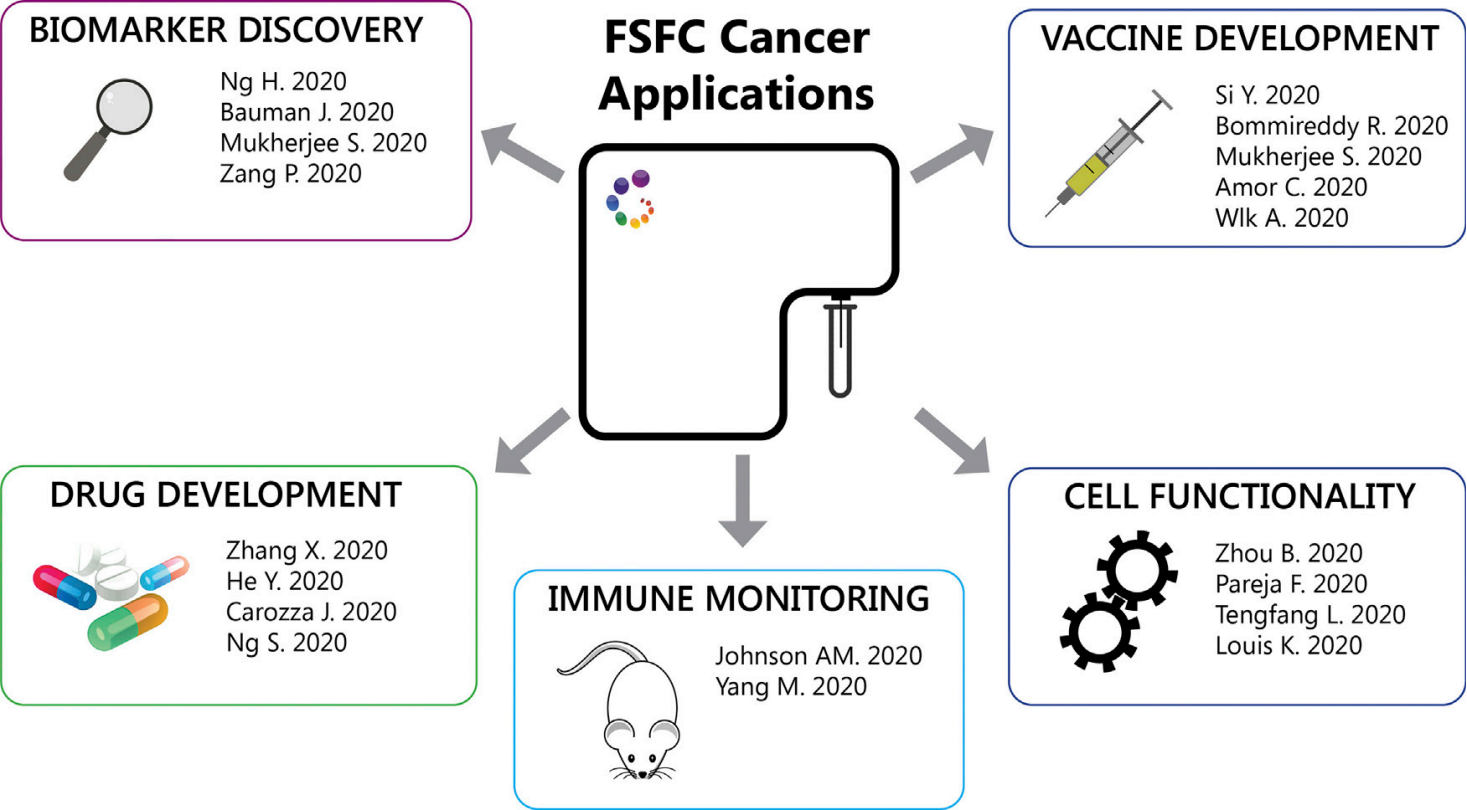
FSFC applications. Through the characterization of the immune cell response, FSFC has enable multiple applications: characterization of tumor immune infiltrates, biomarker exploration, evaluation of new drug and vaccine targets and testing of cell functionality.

Other FSFC applications described so far are: 1. Detection of mitochondria reactive oxygen species to evaluate mitochondrial respiration of hospitalized heart failure patients. Heart failure was associated with reduced respiratory capacity and elevated proinflammatory cytokine responses (Zhou et al., 2020). 2. Detection of lysosomal activity using a fluorescent substrate, to evaluate the role of specific mutations in granular cell tumors. Granular cell tumors are rare tumors characterized by abundant intracytoplasmic granules. The authors identified inactivating somatic mutations in endosomal pH regulators causing this type of tumors. Gene silencing impaired vesicle acidification, redistribution of endosomal compartments and accumulation of intracytoplasmic granules. In addition, depletion of these regulators results in the acquisition of oncogenic properties (Pareja et al., 2018). 3. Detection of nitric oxygen species in macrophages after heart transplantation. IL-33 restricts the proinflammatory capacity of graft-infiltrating macrophages after heart transplantation preventing the subsequent transplant rejection (Li et al., 2020). 4. Detection of intracellular cytokines to characterize IL-21 production in T follicular helper (T_FH_) cells in patients undergoing antibody-mediated rejection after kidney transplantation These circulating IL-21-producing T_FH_ cells also expressed higher levels of the checkpoint receptors ICOS and PD-1, memory markers CCR7 CD127 subsets and transcription factors IRF4 and c-Maf (Louis et al., 2020). Figure 2 summarizes the FSFC applications in cancer immunotherapy described so far.

Finally, since COVID-19 research has been the center of attention this year, a few studies have addressed the evaluation of immunological responses against this disease using FSFC. Severe symptoms correlated with low frequency of non-classical CD14^Low^CD16^High^ monocytes, accumulation of HLA-DR^Low^ classical monocytes and calprotectin released by immature CD10^Low^CD101^-^CXCR4^+/-^ suppressive neutrophils (Silvin et al., 2020). COVID-19 patients mildly symptomatic developed SARS-CoV-2-specific antibodies and had virus-specific memory B and T cells. The virus-specific memory lymphocytes secreted IFN-γ and proliferate and memory B cells expressed receptors capable of neutralizing virus, suggesting functional features of antiviral protective immunity (Rodda et al., 2021).

## Challenges in the Implementation of Flow Cytometry for Immune Monitoring

The use of any type of cytometry technology for longitudinal cancer immunotherapy studies requires reproducible protocols with careful standardization of every step in the workflow, from experimental and panel design to sample handling/acquisition and data analysis, to obtain reliable results (Laskowski et al., 2020). Multiple challenges can be faced during the implementation of cytometry in immune monitoring studies: gating and analysis subjectivity; operator level of training; instrument maintenance and QC; lack of protocol consistency; lack of optimized controls; and technical errors during staining, acquisition or data analysis and interpretation. The informed selection of reagents and controls, together with careful panel and experimental design and proficiency in instrument operation, will minimize data variability. Reproducibility can also be achieved using automated equipment for sample preparation and staining, as well as machine learning tools for data quality assessment, cleaning, transformation, batch effect identification and normalization, clustering, visualization, predictive statistical analysis and biomarker discovery (White et al., 2014).

## Guidelines to Generate FSFC High Quality Data

High-quality data means that dim or rare cell subsets can be easily resolved, and that all populations of interest can be visualized and easily gated by the investigator. As with any other technology, careful experiment and panel design are required to generate high quality data. FSFC panel design requires prior knowledge of the biology of the populations in the assay, the cytometer optical configuration, the levels of expression of the proteins in the panel and the fluorescence emission signatures of the selected fluorophores. Careful optimization of the assay is also required, including sample preparation, antibody titration, Fc receptor blocking, reference control selection, incorporation of biological positive controls, validation of the staining protocol and optimization of acquisition settings. The publication by Ferrer-Font et al. described a simple protocol to follow for successful design and optimization of a FSFC panel (Ferrer-Font et al., 2020b). Other publications provide additional guidelines (Park et al., 2020; Solomon et al., 2020). Park et al. designed the first 40-color panel for identification of multiple hematopoietic cells, including T cells, NK cells, B cells, monocytes and dendritic cells for immunological studies, describing in details the step for successful panel design. For each specific cell type, the panel includes markers for further characterization, such as activation and differentiation markers and chemokine receptors. (Park et al., 2020).

**FIGURE 3:**
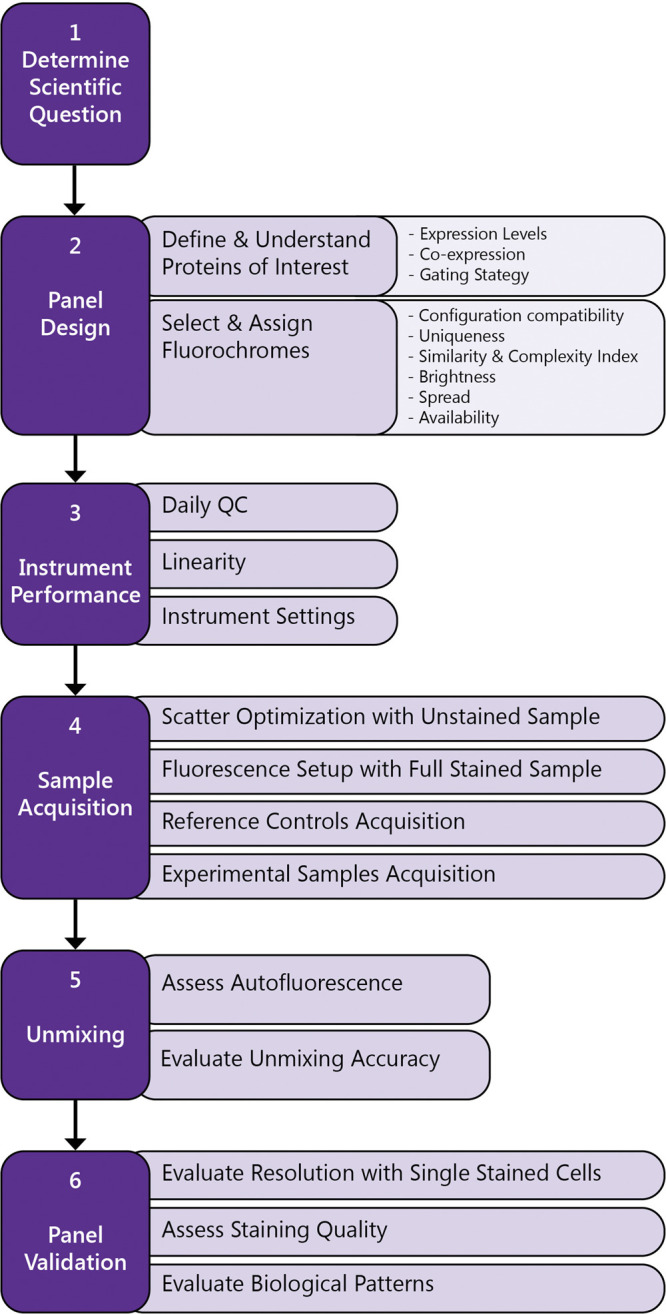
Workflow to generate high quality FSFC data.

Here the most important considerations for a successful experiment using FSFC. A simplified workflow is described in Figure 3.

1. Formulate the experimental question and confirm FSFC will provide the correct readout with optimal resolution.
2. List the proteins of interest, their antigen density and their co-expression levels. Select the antibody clones and reagents using validated antibodies and based on previous literature.
3. Define the theoretical gating strategy to be used when testing the panel.
4. Select fluorochromes with unique spectral signatures based on your instrument configuration.
5. Assign a fluorochrome to each marker based on antigen density and dye brightness, uniqueness, spread and availability.
6. Guide fluorochrome assignment by using Similarity Index and Complexity Index to evaluate overall similarity for the selected fluorochromes and Cross Stain Index matrix to evaluate spread impact between the selected fluorophores.
7. Verify instrument performance by running daily QC.
8. Use Cytek Assay Setting (CAS) for sample acquisition as a starting point. CAS provide optimized instrument settings to maximize resolution, minimize spread and ensure the uniqueness and accuracy of the spectral emission profiles. CAS are adjusted automatically during daily QC using calibration beads.
9. Optimize FSC and SSC gains and threshold to have your population of interest on scale.
10. Acquire all your samples using the same instrument settings (gain, threshold, signal type, area scaling factor and laser delays).
11. Check the fully stained sample first. If the signal is off scale, titration is recommended, but gains can be adjusted to allow brighter signals to be detected accurately.
12. Acquire an unstained control and one single-color control per fluorochrome in the panel.
13. QC the reference controls by confirming the accuracy of the spectral signature and ruling out spectral mismatch, contamination, reagent degradation or carryover between samples.
14. Unmix the data using the reference controls to determine the contribution of each fluorochrome to the fully stained samples.
15. Determine if autofluorescence extraction is needed, based on the emission profile observed in the unstained control and its impact on fluorescence resolution for the fluorochromes in the panel.
16. Validate the panel using single stained cells. Compare intensity of fluorescence in the positive population and spread in the negative population, in single stain sample versus the multicolor sample. Identify loss of staining resolution and take corrective actions if needed, reassigning fluorochromes, modifying the staining protocol or changing the antibody concentration. If the resolution and the assay readout are not highly impacted, then the panel can be used.

Reference controls (RCs) are the single stained controls used for unmixing calculation. The recommendations to prepare high quality RCs are:

- They should be as bright or brighter than the fluorescence in the fully stained samples.
- The autofluorescence of the events in the negative and positive peaks should be similar. In other words, use the same type of particle for both populations.
- They should be treated the same way as the multicolor samples, including the same washes, incubations, fixation, permeabilizations or any step that could alter the emission profile.
- Record enough events for the unmixing calculation and for the software to clearly identify the spectral signatures, having at least 500 events in each interval gate.
- Both compensation beads and cells can be used as RC. Cells are the ideal control to match the dye spectral profile in the multicolor samples, but beads are a good option for low density antigens or rare populations.

Similar single control recommendations also apply for conventional flow cytometry and have been described before (Ashhurst et al., 2017). In FSFC, an unstained control is always necessary for every experiment, for both unmixing and autofluorescence extraction. The unstained cells should match the cell type and staining protocol used for the fully stained sample. Multiple unstained controls can be recorded to match exactly each stimulation condition, treatment or sample type.

## Conclusion

Immune monitoring is critical to correlate immune signatures with cancer progression in preclinical studies and clinical trials. Cytometry technologies allow us to identify the type of immune cells present, as well as, features about their function, maturation, activation or memory status. The utmost goal is to use these cell immune profiles as biomarkers to identify successful treatments and guide therapy decisions for cancer patients. The clinical applications of cytometry in oncology started with DNA cell cycle profiling as a prognostic tool, and then expanded, using fluorochrome-conjugated antibodies, to a broad range of applications: diagnosis of hematological malignancies, study of minimal residual disease, quantification of tumor circulating cells and cancer immune surveillance after treatment. Clinical biomarkers are still needed to characterize new therapies, to understand the mechanisms behind treatment resistance and to tailor personalized treatment.

FSFC has further enabled high throughput multi-parametric characterization of single cells in suspension for immune evaluations of up to 40 parameters in the same sample. This technology combines high sensitivity, empowering new applications with reagent flexibility and better resolution and expanding the accessibility through lower cost. The higher sensitivity of this technology contributes to generating higher quality high-dimensional immune characterizations critical for biomarker discovery. Since more markers can be evaluated simultaneously, FSFC addresses the issues of limited sample availability by increasing the amount of information obtained from a single sample. The use of FSFC in cancer immune surveillance has facilitated the identification of biomarkers, exploration of immune correlates in immunotherapy trials and for new therapies and the characterization of cell phenotype and functionality, measuring cell proliferation, cytotoxicity or cytokine production. The use of this technology has facilitated in-depth characterizations of multiple immune cell subpopulations for the IO field with very promising results and supported by multiple investigations.

Generation of FSFC high quality results, to support immunotherapy clinical studies requires reliable data and involves many factors that could impact the robustness of the studies. Panel design and validation, sample preparation, antibody titration, optimized reference controls, standardized staining protocols, inclusion of biological controls for normalization, inclusion of controls for gating purposes and instrument quality control need to be included into the experimental workflow. Similarly, optimized instrument settings and correction for variation in instrument performance are required to render reproducible results with optimal population resolution. Spectral cytometry is a powerful tool available for immune monitoring in cancer immunotherapy studies and clinical trials. The successful implementation of any cytometry technology relies on monitoring the quality of the process and the data, to ensure robustness and reproducibility in any scientific conclusion drawn from the observations.

## Author Contributions

DB wrote the manuscript and both GR and EC contributed in the reviewing process with suggestions and editing. EC was the person invited by your journal to write this manuscript originally, but since DB has experience in the cancer immune monitoring field she was the lead person.

## Conflict of Interest

The three authors worked for the company that developed the full spectrum flow cytometry technology. We were asked to write this review by the editor to show the contributions to the immunoncology field.

## References

[B1] Allison J. P. (2015). Checkpoints. Cell 162 (6), 1202–1205. 10.1016/j.cell.2015.08.047 26359978

[B2] Amor C. Feucht J. Leibold J. Ho Y.-J. Zhu C. Alonso-Curbelo D. (2020). Senolytic CAR T cells reverse senescence-associated pathologies. Nature 583 (7814), 127–132. 10.1038/s41586-020-2403-9 32555459 PMC7583560

[B3] Ashhurst T. M. Smith A. L. King N. J. C. (2017). High-dimensional fluorescence cytometry. Curr. Protocols Immunol. 119, 1–38. 10.1002/cpim.37 29091263

[B4] Barone S. M. Paul A. G. A. Muehling L. M. Lannigan J. A. Kwok W. W. Turner R. B. (2020). Unsupervised machine learning reveals key immune cell subsets in COVID-19, rhinovirus infection, and cancer therapy. bioRxiv. 10.1101/2020.07.31.190454 PMC8370768 34350827

[B5] Basiji D. A. (2016). Principles of amnis imaging flow cytometry. Methods Mol. Biol. 1389, 13–21. 10.1007/978-1-4939-3302-0_2 27460235

[B6] Bauman J. E. Ohr J. Gooding W. E. Ferris R. L. Duvvuri U. Kim S. (2020). Phase I study of ficlatuzumab and cetuximab in cetuximab-resistant, recurrent/metastatic head and neck cancer. Cancers 12 (6), 1537 10.3390/cancers12061537 PMC7352434 32545260

[B7] Bendall S. C. Nolan G. P. (2012). From single cells to deep phenotypes in cancer. Nat. Biotechnol. 30 (7), 639–647. 10.1038/nbt.2283 22781693

[B8] Bommireddy R. Munoz L. E. Kumari A. Huang L. Fan Y. Monterroza L. (2020). Tumor membrane vesicle vaccine augments the efficacy of anti-PD1 antibody in immune checkpoint inhibitor-resistant squamous cell carcinoma models of head and neck cancer. Vaccine 8 (2), 182 10.3390/vaccines8020182 PMC7348725 32295135

[B9] Carozza J. A. Böhnert V. Nguyen K. C. Skariah G. Shaw K. E. Brown J. A. (2020). Extracellular cGAMP is a cancer-cell-produced immunotransmitter involved in radiation-induced anticancer immunity. Nat. Cancer 1 (2), 184–196. 10.1038/s43018-020-0028-4 PMC7990037 33768207

[B10] Cossarizza A. Chang H. D. Radbruch A. Acs A. Adam D. Adam-Klages S. (2019). Guidelines for the use of flow cytometry and cell sorting in immunological studies (second edition). Eur. J. Immunol. 49 (10), 1457–1973. 10.1002/eji.201970107 31633216 PMC7350392

[B11] Cunningham R. A. Holland M. McWilliams E. Hodi F. S. Severgnini M. (2019). Detection of clinically relevant immune checkpoint markers by multicolor flow cytometry. J. Biol. Methods 6 (2), 114 10.14440/jbm.2019.283 PMC6706095 31453261

[B12] Ferrer-Font L. Mayer J.U. Old S. Hermans I.F. Irish J. Price K.M. (2020a). High-dimensional data analysis algorithms yield comparable results for mass cytometry and spectral flow cytometry data. Cytometry A. 97 (8), 824–831. 10.1002/cyto.a.24016 32293794 PMC7682594

[B13] Ferrer-Font L. Pellefigues C. Mayer J. U. Small S. J. Jaimes M. C. Price K. M. (2020b). Panel design and optimization for high-dimensional immunophenotyping assays using spectral flow cytometry. Curr. Protoc. Cytom. 92 (1), e70 10.1002/cpcy.70 32150355

[B14] Fox A. Dutt T. S. Karger B. Obregón-Henao A. Anderson G. B. Henao-Tamayo M. (2020). Acquisition of high‐quality spectral flow cytometry data. Curr. Protoc. Cytom. 93 (1), e74 10.1002/cpcy.74 32421215 PMC8801208

[B15] Fox B. A. Schendel D. J. Butterfield L. H. Aamdal S. Allison J. P. Ascierto P. A. (2011). Defining the critical hurdles in cancer immunotherapy. J. Transl. Med. 9, 214 10.1186/1479-5876-9-214 22168571 PMC3338100

[B16] Guo Q. Huang F. Goncalves C. del Rincón S. V. Miller W. H. (2019). Translation of cancer immunotherapy from the bench to the bedside. Adv. Cancer Res. 143, 1–62. 10.1016/bs.acr.2019.03.001 31202357

[B17] He Y. Koch R. Budamagunta V. Zhang P. Zhang X. Khan S. (2020). DT2216-a Bcl-xL-specific degrader is highly active against Bcl-xL-dependent T cell lymphomas. J. Hematol. Oncol. 13 (1), 95 10.1186/s13045-020-00928-9 32677976 PMC7364785

[B18] Heel K. Tabone T. Röhrig K. J. Maslen P. G. Meehan K. Grimwade L. F. (2013). Developments in the immunophenotypic analysis of haematological malignancies. Blood Rev. 27 (4), 193–207. 10.1016/j.blre.2013.06.005 23845589

[B19] Hodi F. S. O'Day S. J. McDermott D. F. Weber R. W. Sosman J. A. Haanen J. B. (2010). Improved survival with ipilimumab in patients with metastatic melanoma. N. Engl. J. Med. 363 (8), 711–723. 10.1056/NEJMoa1003466 20525992 PMC3549297

[B20] Irish J. M. Doxie D. B. (2014). High-dimensional single-cell cancer biology. Curr. Top. Microbiol. Immunol. 377, 1–21. 10.1007/82_2014_367 24671264 PMC4216808

[B21] Johnson A. M. Bullock B. L. Neuwelt A. J. Poczobutt J. M. Kaspar R. E. Li H. Y. (2020). Cancer cell-intrinsic expression of MHC class II regulates the immune microenvironment and response to Anti-PD-1 therapy in lung adenocarcinoma. J. Immunol. 204 (8), 2295–2307. 10.4049/jimmunol.1900778 32179637 PMC7472648

[B22] Laskowski T. J. Hazen A. L. Collazo R. S. Haviland D. (2020). Rigor and reproducibility of cytometry practices for immuno-oncology: a multifaceted challenge. Cytom. A. 97 (2), 116–125. 10.1002/cyto.a.23882 31454153

[B23] Li T. Zhang Z. Bartolacci J. G. Dwyer G. K. Liu Q. Mathews L. R. (2020). Graft IL-33 regulates infiltrating macrophages to protect against chronic rejection. J. Clin. Investig. 130(10), 5397–5412. 10.1172/JCI133008 32644975 PMC7524467

[B24] Louis K. Macedo C. Bailly E. Lau L. Ramaswami B. Marrari M. (2020). Coordinated circulating T follicular helper and activated B cell responses underlie the onset of antibody-mediated rejection in kidney transplantation. J. Am. Soc. Nephrol. 31 (10), 2457–2474. 10.1681/ASN.2020030320 32723838 PMC7608995

[B25] Maecker H. T. Harari A. (2015). Immune monitoring technology primer: flow and mass cytometry. J. Immunother. Cancer 3, 44 10.1186/s40425-015-0085-x 26380089 PMC4570613

[B26] Mattiuzzi C. Lippi G. (2019). Current cancer epidemiology. JEGH. 9 (4), 217–222. 10.2991/jegh.k.191008.001 31854162 PMC7310786

[B27] Mukherjee S. Lanfredini S. Cox C. Thapa A. Hughes S. Bangs F. (2020). Translational analysis from SCALOP trial: CCL5 as a prognostic biomarker and a potentially actionable target in locally advanced pancreatic cancer (LAPC). Alexandria , VA: American Society of Clinical Oncology.

[B28] Ng H. H. M. Lee R. Y. Goh S. Tay I. S. Y. Lim X. Lee B. (2020). Immunohistochemical scoring of CD38 in the tumor microenvironment predicts responsiveness to anti-PD-1/PD-L1 immunotherapy in hepatocellular carcinoma. J. Immunother. Cancer 8 (2), e000987 10.1136/jitc-2020-000987 32847986 PMC7451957

[B29] Niewold P. Ashhurst T. M. Smith A. L. King N. J. C. (2020). Evaluating spectral cytometry for immune profiling in viral disease. Cytom. A. 97, 1165 10.1002/cyto.a.24211 32799382

[B30] Nolan J. P. Condello D. (2013). Spectral flow cytometry. Curr. Protoc. Cytom. 63, 28 10.1002/0471142956.cy0127s63 PMC3556726 23292705

[B31] Nolan J. P. Condello D. Duggan E. Naivar M. Novo D. (2013). Visible and near infrared fluorescence spectral flow cytometry. Cytom. A. 83A (3), 253–264. 10.1002/cyto.a.22241 PMC3594514 23225549

[B32] Pareja F. Brandes A. H. Basili T. Selenica P. Geyer F. C. Fan D. (2018). Loss-of-function mutations in ATP6AP1 and ATP6AP2 in granular cell tumors. Nat. Commun. 9 (1), 3533 10.1038/s41467-018-05886-y 30166553 PMC6117336

[B33] Park L. M. Lannigan J. Jaimes M. C. (2020). OMIP-069: Forty-color full spectrum flow cytometry panel for deep immunophenotyping of major cell subsets in human peripheral blood. Cytom. A, 97, 1044 10.1002/cyto.a.24213 PMC8132182 32830910

[B35] Robinson J. P. (2004). Multispectral cytometry: the next generation. Biophotonics Int. 11, 36–41. 10.1002/cyto.a.20889

[B36] Robinson J. P. (2019). Spectral flow cytometry- Quo vadimus ? Cytom. A. 95 (8), 823–824. 10.1002/cyto.a.23779 31038271

[B53] Rodda L. B. Netland J. Shehata L. Pruner K. B. Morawski P. A. Thouvenel C. D. (2021). Functional SARS-CoV-2-specific immune memory persists after mild COVID-19. Cell. 184 (1), 169–183. 10.1016/j.cell.2020.11.029 33296701 PMC7682481

[B37] Sharma P. Allison J. P. (2015). Immune checkpoint targeting in cancer therapy: toward combination strategies with curative potential. Cell 161 (2), 205–214. 10.1016/j.cell.2015.03.030 25860605 PMC5905674

[B38] Sharma P. Wagner K. Wolchok J. D. Allison J. P. (2011). Novel cancer immunotherapy agents with survival benefit: recent successes and next steps. Nat. Rev. Cancer 11 (11), 805–812. 10.1038/nrc3153 22020206 PMC3426440

[B39] Shissler S. C. Singh N. J. Webb T. J. (2020). thymic resident nKt cell subsets show differential requirements for CD28 co-stimulation during antigenic activation. Sci. Rep. 10 (1), 1–13. 10.1038/s41598-020-65129-3 32427927 PMC7237672

[B40] Si Y. Tian Q. Zhao F. Kelly S. H. Shores L. S. Camacho D. F. (2020). Adjuvant-free nanofiber vaccine induces in situ lung dendritic cell activation and TH17 responses. Sci. Adv. 6 (32), eaba0995 10.1126/sciadv.aba0995 32821819 PMC7413739

[B41] Silvin A. Chapuis N. Dunsmore G. Goubet A.-G. Dubuisson A. Derosa L. (2020). Elevated calprotectin and abnormal myeloid cell subsets discriminate severe from Mild COVID-19. Cell 182 (6), 1401–1418. 10.1016/j.cell.2020.08.002 32810439 PMC7405878

[B42] Solomon M. DeLay M. Reynaud D. (2020). Phenotypic analysis of the mouse hematopoietic hierarchy using spectral cytometry: from stem cell subsets to early progenitor compartments. Cytom. A. 97 (10), 1057–1065. 10.1002/cyto.a.24041 PMC8630828 32449586

[B43] Spitzer M. H. Nolan G. P. (2016). Mass cytometry: single cells, many features. Cell 165 (4), 780–791. 10.1016/j.cell.2016.04.019 27153492 PMC4860251

[B44] Stoeckius M. Hafemeister C. Stephenson W. Houck-Loomis B. Chattopadhyay P. K. Swerdlow H. (2017). Simultaneous epitope and transcriptome measurement in single cells. Nat. Methods 14 (9), 865–868. 10.1038/nmeth.4380 28759029 PMC5669064

[B45] Wei S. C. Duffy C. R. Allison J. P. (2018). Fundamental mechanisms of immune checkpoint blockade therapy. Cancer Discov. 8 (9), 1069–1086. 10.1158/2159-8290.CD-18-0367 30115704

[B46] White S. Laske K. Welters M. J. Bidmon N. van der Burg S. H. Britten C. M. (2014). Managing multi-center flow cytometry data for immune monitoring. Cancer Inf. 13 (Suppl 7), 111–122. 10.4137/CIN.S16346 PMC4463798 26085786

[B47] Wilk A. J. Weidenbacher N. L.-B. Vergara R. Haabeth O. A. W. Levy R. Waymouth R. M. (2020). Charge-altering releasable transporters enable phenotypic manipulation of natural killer cells for cancer immunotherapy. Blood Adv. 4 (17), 4244–4255. 10.1182/bloodadvances.2020002355 32898247 PMC7479957

[B48] Yang M. Du W. Yi L. Wu S. He C. Zhai W. (2020). Checkpoint molecules coordinately restrain hyperactivated effector T cells in the tumor microenvironment. Oncoimmunology 9 (1), 1708064 10.1080/2162402X.2019.1708064 32076578 PMC6999836

[B49] Yuan J. Hegde P. S. Clynes R. Foukas P. G. Harari A. Kleen T. O. (2016). Novel technologies and emerging biomarkers for personalized cancer immunotherapy. J. Immunother. Cancer 4, 3 10.1186/s40425-016-0107-3 26788324 PMC4717548

[B50] Zhang X. Thummuri D. Liu X. Hu W. Zhang P. Khan S. (2020). Discovery of PROTAC BCL-XL degraders as potent anticancer agents with low on-target platelet toxicity. Eur. J. Med. Chem. 192, 112186 10.1016/j.ejmech.2020.112186 32145645 PMC7433031

[B51] Zhou B. Wang D. D. Qiu Y. Airhart S. Liu Y. Stempien-Otero A. (2020). Boosting NAD level suppresses inflammatory activation of PBMC in heart failure. J. Clin. Invest. 130 (11), 6054–6063. 10.1172/JCI138538 32790648 PMC7598081

